# Supplementary material for: Development of innovative multi-epitope mRNA vaccine against central nervous system tuberculosis using in silico approaches
Source: PLoS One. 2024 Sep 6;19(9):e0307877. doi: 10.1371/journal.pone.0307877 (PMC11379207; doi:10.1371/journal.pone.0307877)
Supplement: S1 Table — (DOCX) [file pone.0307877.s001.docx]

**PLOS ONE**

**Article title:Development of innovative multi-epitope mRNA vaccine against central nervous system tuberculosis using in silico approaches**

**Author:Huidong Shi**

**S1 Table. MHC-I Binding Prediction Results of Rv0986(IEDB)**

| Allele | start | end | peptide | Score | Percentile Rank |
| --- | --- | --- | --- | --- | --- |
| HLA-A*11:01 | 125 | 133 | VVARDLLEK | 0.851532 | 0.05 |
| HLA-A*11:01 | 239 | 247 | STILLPTSY | 0.601441 | 0.21 |
| HLA-A*11:01 | 114 | 122 | ELAGVSQRK | 0.181509 | 0.95 |
| HLA-A*11:01 | 203 | 211 | ATHSPSMTQ | 0.151101 | 1.10 |
| HLA-A*11:01 | 215 | 223 | RVVNLQGGR | 0.145681 | 1.20 |
| HLA-A*11:01 | 9 | 17 | LSNLSWTFR | 0.108557 | 1.40 |
| HLA-A*11:01 | 146 | 154 | KLSGGEQQR | 0.082704 | 1.60 |
| HLA-A*11:01 | 41 | 49 | LLGQSGSGK | 0.063879 | 1.80 |
| HLA-A*11:01 | 14 | 22 | WTFREGETR | 0.063325 | 1.80 |
| HLA-A*11:01 | 69 | 77 | INGFAITQK | 0.040725 | 2.30 |

| Allele | start | end | peptide | Score | Percentile Rank |
| --- | --- | --- | --- | --- | --- |
| HLA-A*02:01 | 160 | 168 | ALAHNPMLV | 0.837155 | 0.06 |
| HLA-A*02:01 | 98 | 106 | NLIPTLTVL | 0.702799 | 0.13 |
| HLA-A*02:01 | 110 | 118 | TLPQELAGV | 0.701237 | 0.13 |
| HLA-A*02:01 | 188 | 196 | VLLDLTRQA | 0.650501 | 0.16 |
| HLA-A*02:01 | 184 | 192 | KVLDVLLDL | 0.643122 | 0.17 |
| HLA-A*02:01 | 51 | 59 | TLLNLISGI | 0.514178 | 0.26 |
| HLA-A*02:01 | 201 | 209 | IMATHSPSM | 0.445762 | 0.32 |
| HLA-A*02:01 | 25 | 33 | VLDHITFDF | 0.10928 | 1.30 |
| HLA-A*02:01 | 95 | 103 | QFFNLIPTL | 0.103423 | 1.40 |
| HLA-A*02:01 | 208 | 216 | SMTQHADRV | 0.089855 | 1.50 |

| Allele | start | end | peptide | Score | Percentile Rank |
| --- | --- | --- | --- | --- | --- |
| HLA-A*03:01 | 125 | 133 | VVARDLLEK | 0.730703 | 0.14 |
| HLA-A*03:01 | 41 | 49 | LLGQSGSGK | 0.425441 | 0.44 |
| HLA-A*03:01 | 146 | 154 | KLSGGEQQR | 0.271061 | 0.72 |
| HLA-A*03:01 | 239 | 247 | STILLPTSY | 0.205579 | 0.91 |
| HLA-A*03:01 | 215 | 223 | RVVNLQGGR | 0.193177 | 0.96 |
| HLA-A*03:01 | 114 | 122 | ELAGVSQRK | 0.193049 | 0.96 |
| HLA-A*03:01 | 203 | 211 | ATHSPSMTQ | 0.086449 | 1.70 |
| HLA-A*03:01 | 155 | 163 | VAISRALAH | 0.057657 | 2.10 |
| HLA-A*03:01 | 69 | 77 | INGFAITQK | 0.033112 | 2.70 |
| HLA-A*03:01 | 9 | 17 | LSNLSWTFR | 0.027721 | 3.00 |
