# Supplementary material for: Development of innovative multi-epitope mRNA vaccine against central nervous system tuberculosis using in silico approaches
Source: PLoS One. 2024 Sep 6;19(9):e0307877. doi: 10.1371/journal.pone.0307877 (PMC11379207; doi:10.1371/journal.pone.0307877)
Supplement: S2 Table — (DOCX) [file pone.0307877.s002.docx]

**PLOS ONE**

**Article title:Development of innovative multi-epitope mRNA vaccine against central nervous system tuberculosis using in silico approaches**

**Author:Huidong Shi**

**S2 Table. MHC-I Binding Prediction Results of Rv0986(NetCTLpan-1.1)**

| Allele | start | end | peptide | Score | Percentile Rank |
| --- | --- | --- | --- | --- | --- |
| HLA-A*11:01 | 239 | 247 | STILLPTSY | 0.85728 | 0.8 |
| HLA-A*11:01 | 125 | 133 | VVARDLLEK | 0.83438 | 0.8 |
| HLA-A*11:01 | 9 | 17 | LSNLSWTFR | 0.75170 | 1.5 |
| HLA-A*11:01 | 114 | 122 | ELAGVSQRK | 0.49209 | 5.0 |
| HLA-A*11:01 | 41 | 49 | LLGQSGSGK | 0.48579 | 5.0 |
| HLA-A*11:01 | 69 | 77 | INGFAITQK | 0.46583 | 5.0 |
| HLA-A*11:01 | 53 | 61 | LNLISGIEK | 0.43309 | 6.0 |
| HLA-A*11:01 | 14 | 22 | WTFREGETR | 0.42258 | 6.0 |
| HLA-A*11:01 | 215 | 223 | RVVNLQGGR | 0.41787 | 6.0 |
| HLA-A*11:01 | 92 | 100 | IVFQFFNLI | 0.39455 | 7.0 |

| Allele | start | end | peptide | Score | Percentile Rank |
| --- | --- | --- | --- | --- | --- |
| HLA-A*02:01 | 160 | 168 | ALAHNPMLY | 0.93988 | 0.8 |
| HLA-A*02:01 | 51 | 59 | TLLNLISGI | 0.90200 | 0.8 |
| HLA-A*02:01 | 110 | 118 | TLPQELAGY | 0.88169 | 0.8 |
| HLA-A*02:01 | 201 | 209 | IMATHSPSM | 0.81968 | 1.0 |
| HLA-A*02:01 | 184 | 192 | KVLDVLLDL | 0.78335 | 1.5 |
| HLA-A*02:01 | 98 | 106 | NLIPTLTVL | 0.77390 | 1.5 |
| HLA-A*02:01 | 94 | 102 | FQFFNLIPT | 0.65832 | 3.0 |
| HLA-A*02:01 | 188 | 196 | VLLDLTRQA | 0.64242 | 3.0 |
| HLA-A*02:01 | 92 | 100 | IVFQFFNLI | 0.63955 | 3.0 |
| HLA-A*02:01 | 25 | 33 | VLDHITFDF | 0.60336 | 4.0 |

| Allele | start | end | peptide | Score | Percentile Rank |
| --- | --- | --- | --- | --- | --- |
| HLA-A*03:01 | 125 | 133 | VVARDLLEK | 0.63538 | 1.5 |
| HLA-A*03:01 | 41 | 49 | LLGQSGSGK | 0.60479 | 2.0 |
| HLA-A*03:01 | 239 | 247 | STILLPTSY | 0.57628 | 2.0 |
| HLA-A*03:01 | 114 | 122 | ELAGVSQRK | 0.48209 | 3.0 |
| HLA-A*03:01 | 9 | 17 | LSNLSWTFR | 0.45370 | 4.0 |
| HLA-A*03:01 | 146 | 154 | KLSGGEQQR | 0.43085 | 4.0 |
| HLA-A*03:01 | 201 | 209 | IMATHSPSM | 0.40068 | 5.0 |
| HLA-A*03:01 | 8 | 16 | QLSNLSWTF | 0.39738 | 5.0 |
| HLA-A*03:01 | 160 | 168 | ALAHNPMLV | 0.39388 | 5.0 |
| HLA-A*03:01 | 69 | 77 | INGFAITQK | 0.38583 | 6.0 |
