# Supplementary material for: Development of innovative multi-epitope mRNA vaccine against central nervous system tuberculosis using in silico approaches
Source: PLoS One. 2024 Sep 6;19(9):e0307877. doi: 10.1371/journal.pone.0307877 (PMC11379207; doi:10.1371/journal.pone.0307877)
Supplement: S3 Table — (DOCX) [file pone.0307877.s003.docx]

**PLOS ONE**

**Article title:Development of innovative multi-epitope mRNA vaccine against central nervous system tuberculosis using in silico approaches**

**Author:Huidong Shi**

**S3 Table. MHC-I Binding Prediction Results of PknD(IEDB)**

| Allele | start | end | peptide | Score | Percentile Rank |
| --- | --- | --- | --- | --- | --- |
| HLA-A*11:01 | 97 | 105 | GTSLRALLK | 0.824076 | 0.06 |
| HLA-A*11:01 | 54 | 62 | AVFRARMQR | 0.803221 | 0.08 |
| HLA-A*11:01 | 248 | 256 | QVIAKGMAK | 0.802293 | 0.08 |
| HLA-A*11:01 | 395 | 403 | AAAGYLVLR | 0.624762 | 0.19 |
| HLA-A*11:01 | 441 | 449 | VTSEGMYGR | 0.541079 | 0.26 |
| HLA-A*11:01 | 93 | 101 | RMIDGTSLR | 0.537469 | 0.26 |
| HLA-A*11:01 | 368 | 376 | HAVPPAGNK | 0.523548 | 0.28 |
| HLA-A*11:01 | 172 | 180 | GTAVGTYNY | 0.380956 | 0.45 |
| HLA-A*11:01 | 369 | 377 | AVPPAGNKR | 0.292446 | 0.61 |
| HLA-A*11:01 | 461 | 469 | TVLPFNGLY | 0.28957 | 0.61 |

| Allele | start | end | peptide | Score | Percentile Rank |
| --- | --- | --- | --- | --- | --- |
| HLA-A*02:01 | 445 | 453 | GMYGRVVKL | 0.92855 | 0.03 |
| HLA-A*02:01 | 425 | 433 | RLSPSGVAV | 0.740365 | 0.11 |
| HLA-A*02:01 | 483 | 491 | YVTDFNNRV | 0.585315 | 0.21 |
| HLA-A*02:01 | 105 | 113 | KQYGPLTPA | 0.448042 | 0.32 |
| HLA-A*02:01 | 10 | 18 | SQFGPYQLL | 0.444322 | 0.32 |
| HLA-A*02:01 | 262 | 270 | FMSAGDLAI | 0.423159 | 0.34 |
| HLA-A*02:01 | 241 | 249 | RVPPALDQV | 0.415 | 0.35 |
| HLA-A*02:01 | 365 | 373 | SLGHAVPPA | 0.410774 | 0.35 |
| HLA-A*02:01 | 350 | 358 | SQTGHSPAV | 0.375074 | 0.4 |
| HLA-A*02:01 | 593 | 601 | ITAPWGIAV | 0.289413 | 0.57 |

| Allele | start | end | peptide | Score | Percentile Rank |
| --- | --- | --- | --- | --- | --- |
| HLA-A*03:01 | 54 | 62 | AVFRARMQR | 0.770693 | 0.11 |
| HLA-A*03:01 | 93 | 101 | RMIDGTSLR | 0.769951 | 0.11 |
| HLA-A*03:01 | 97 | 105 | GTSLRALLK | 0.768499 | 0.11 |
| HLA-A*03:01 | 248 | 256 | QVIAKGMAK | 0.767824 | 0.11 |
| HLA-A*03:01 | 368 | 376 | HAVPPAGNK | 0.460762 | 0.4 |
| HLA-A*03:01 | 42 | 50 | ALKLISPQY | 0.453585 | 0.4 |
| HLA-A*03:01 | 533 | 541 | VVKLAAGSK | 0.383174 | 0.52 |
| HLA-A*03:01 | 99 | 107 | SLRALLKQY | 0.29889 | 0.66 |
| HLA-A*03:01 | 282 | 290 | HQATTILRR | 0.22056 | 0.87 |
| HLA-A*03:01 | 395 | 403 | AAAGYLVLR | 0.185311 | 0.98 |
