# Supplementary material for: Development of innovative multi-epitope mRNA vaccine against central nervous system tuberculosis using in silico approaches
Source: PLoS One. 2024 Sep 6;19(9):e0307877. doi: 10.1371/journal.pone.0307877 (PMC11379207; doi:10.1371/journal.pone.0307877)
Supplement: S4 Table — (DOCX) [file pone.0307877.s004.docx]

**PLOS ONE**

**Article title:Development of innovative multi-epitope mRNA vaccine against central nervous system tuberculosis using in silico approaches**

**Author:Huidong Shi**

**S4 Table. MHC-I Binding Prediction Results of PknD(NetCTLpan-1.1)**

| Allele | start | end | peptide | Score | Percentile Rank |
| --- | --- | --- | --- | --- | --- |
| HLA-A*11:01 | 97 | 105 | GTSLRALLK | 1.00959 | 0.10 |
| HLA-A*11:01 | 54 | 62 | AVFRARMQR | 0.98750 | 0.15 |
| HLA-A*11:01 | 172 | 180 | GTAVGTYNY | 0.84459 | 0.80 |
| HLA-A*11:01 | 93 | 101 | RMIDGTSLR | 0.83718 | 0.80 |
| HLA-A*11:01 | 461 | 469 | TVLPFNGLY | 0.82900 | 0.80 |
| HLA-A*11:01 | 248 | 256 | QVIAKGMAK | 0.79357 | 0.80 |
| HLA-A*11:01 | 395 | 403 | AAAGYLVLR | 0.71454 | 1.50 |
| HLA-A*11:01 | 145 | 153 | LVTASDFAY | 0.60987 | 3.00 |
| HLA-A*11:01 | 207 | 215 | CLTGAPPYR | 0.57868 | 3.00 |
| HLA-A*11:01 | 441 | 449 | VTSEGMYGR | 0.56655 | 4.00 |

| Allele | start | end | peptide | Score | Percentile Rank |
| --- | --- | --- | --- | --- | --- |
| HLA-A*02:01 | 262 | 270 | FMSAGDLAI | 0.97998 | 0.30 |
| HLA-A*02:01 | 445 | 453 | GMYGRVVKL | 0.91414 | 0.80 |
| HLA-A*02:01 | 393 | 401 | IVAAAGYLV | 0.83256 | 1.00 |
| HLA-A*02:01 | 10 | 18 | SQFGPYQLL | 0.80290 | 1.50 |
| HLA-A*02:01 | 425 | 433 | RLSPSGVAV | 0.79528 | 1.50 |
| HLA-A*02:01 | 105 | 113 | KQYGPLTPA | 0.76471 | 1.50 |
| HLA-A*02:01 | 483 | 491 | YVTDFNNRV | 0.73464 | 2.00 |
| HLA-A*02:01 | 593 | 601 | ITAPWGIAV | 0.73124 | 2.00 |
| HLA-A*02:01 | 203 | 211 | VLGECLTGA | 0.71365 | 2.00 |
| HLA-A*02:01 | 151 | 159 | FAYLVDFGI | 0.67896 | 3.00 |

| Allele | start | end | peptide | Score | Percentile Rank |
| --- | --- | --- | --- | --- | --- |
| HLA-A*03:01 | 97 | 105 | GTSLRALLK | 0.86359 | 0.20 |
| HLA-A*03:01 | 54 | 62 | AVFRARMQR | 0.84750 | 0.20 |
| HLA-A*03:01 | 93 | 101 | RMIDGTSLR | 0.80418 | 0.40 |
| HLA-A*03:01 | 42 | 50 | ALKLISPQY | 0.71859 | 0.80 |
| HLA-A*03:01 | 461 | 469 | TVLPFNGLY | 0.66000 | 1.00 |
| HLA-A*03:01 | 172 | 180 | GTAVGTYNY | 0.64859 | 1.50 |
| HLA-A*03:01 | 99 | 107 | SLRALLKQY | 0.63197 | 1.50 |
| HLA-A*03:01 | 248 | 256 | QVIAKGMAK | 0.62457 | 1.50 |
| HLA-A*03:01 | 533 | 541 | VVKLAAGSK | 0.56057 | 2.00 |
| HLA-A*03:01 | 503 | 511 | VLPFDGLNY | 0.48248 | 3.00 |
