# Supplementary material for: Development of innovative multi-epitope mRNA vaccine against central nervous system tuberculosis using in silico approaches
Source: PLoS One. 2024 Sep 6;19(9):e0307877. doi: 10.1371/journal.pone.0307877 (PMC11379207; doi:10.1371/journal.pone.0307877)
Supplement: S5 Table — (DOCX) [file pone.0307877.s005.docx]

**PLOS ONE**

**Article title:Development of innovative multi-epitope mRNA vaccine against central nervous system tuberculosis using in silico approaches**

**Author:Huidong Shi**

**S5 Table. MHC-Ⅱ Binding Prediction Results of Rv0986(IEDB)**

| Allele | start | end | peptide | Score | Percentile Rank |
| --- | --- | --- | --- | --- | --- |
| HLA-DRB1*07:01 | 198 | 212 | KTLIMATHSPSMTQH | 0.7724 | 0.62 |
| HLA-DRB1*07:01 | 197 | 211 | GKTLIMATHSPSMTQ | 0.7371 | 0.84 |
| HLA-DRB1*07:01 | 196 | 210 | AGKTLIMATHSPSMT | 0.5927 | 1.80 |
| HLA-DRB1*07:01 | 94 | 108 | FQFFNLIPTLTVLEN | 0.4086 | 3.70 |
| HLA-DRB1*07:01 | 199 | 213 | TLIMATHSPSMTQHA | 0.3879 | 3.90 |
| HLA-DRB1*07:01 | 151 | 165 | EQQRVAISRALAHNP | 0.3511 | 4.40 |
| HLA-DRB1*07:01 | 93 | 107 | VFQFFNLIPTLTVLE | 0.3169 | 5.20 |
| HLA-DRB1*07:01 | 152 | 166 | QQRVAISRALAHNPM | 0.3081 | 5.40 |
| HLA-DRB1*07:01 | 150 | 164 | GEQQRVAISRALAHN | 0.2948 | 5.70 |
| HLA-DRB1*07:01 | 195 | 209 | QAGKTLIMATHSPSM | 0.2868 | 5.90 |

| Allele | start | end | peptide | Score | Percentile Rank |
| --- | --- | --- | --- | --- | --- |
| HLA-DRB1*03:01 | 75 | 89 | TQKTERDRTLFRRDQ | 0.5452 | 2.30 |
| HLA-DRB1*03:01 | 185 | 199 | VLDVLLDLTRQAGKT | 0.5339 | 2.40 |
| HLA-DRB1*03:01 | 184 | 198 | KVLDVLLDLTRQAGK | 0.4882 | 2.70 |
| HLA-DRB1*03:01 | 74 | 88 | ITQKTERDRTLFRRD | 0.4749 | 2.80 |
| HLA-DRB1*03:01 | 69 | 83 | INGFAITQKTERDRT | 0.4521 | 3.00 |
| HLA-DRB1*03:01 | 68 | 82 | TINGFAITQKTERDR | 0.4237 | 3.30 |
| HLA-DRB1*03:01 | 174 | 188 | TGNLDSDTGDKVLDV | 0.3781 | 3.80 |
| HLA-DRB1*03:01 | 173 | 187 | PTGNLDSDTGDKVLD | 0.3337 | 4.40 |
| HLA-DRB1*03:01 | 70 | 84 | NGFAITQKTERDRTL | 0.3229 | 4.50 |
| HLA-DRB1*03:01 | 76 | 90 | QKTERDRTLFRRDQI | 0.3174 | 4.60 |

| Allele | start | end | peptide | Score | Percentile Rank |
| --- | --- | --- | --- | --- | --- |
| HLA-DRB1*15:01 | 100 | 114 | IPTLTVLENITLPQE | 0.6886 | 0.98 |
| HLA-DRB1*15:01 | 99 | 113 | LIPTLTVLENITLPQ | 0.5509 | 1.70 |
| HLA-DRB1*15:01 | 213 | 227 | ADRVVNLQGGRLIPA | 0.5536 | 1.70 |
| HLA-DRB1*15:01 | 101 | 115 | PTLTVLENITLPQEL | 0.4788 | 2.30 |
| HLA-DRB1*15:01 | 212 | 226 | HADRVVNLQGGRLIP | 0.4196 | 3.10 |
| HLA-DRB1*15:01 | 214 | 228 | DRVVNLQGGRLIPAV | 0.4040 | 3.30 |
| HLA-DRB1*15:01 | 98 | 112 | NLIPTLTVLENITLP | 0.2909 | 5.20 |
| HLA-DRB1*15:01 | 35 | 49 | PGEFVALLGQSGSGK | 0.2775 | 5.50 |
| HLA-DRB1*15:01 | 198 | 212 | KTLIMATHSPSMTQH | 0.2793 | 5.50 |
| HLA-DRB1*15:01 | 221 | 235 | GGRLIPAVNRENQTD | 0.2296 | 6.70 |
