# Supplementary material for: Development of innovative multi-epitope mRNA vaccine against central nervous system tuberculosis using in silico approaches
Source: PLoS One. 2024 Sep 6;19(9):e0307877. doi: 10.1371/journal.pone.0307877 (PMC11379207; doi:10.1371/journal.pone.0307877)
Supplement: S6 Table — (DOCX) [file pone.0307877.s006.docx]

**PLOS ONE**

**Article title:Development of innovative multi-epitope mRNA vaccine against central nervous system tuberculosis using in silico approaches**

**Author:Huidong Shi**

### **S6 Table. MHC-Ⅱ Binding Prediction Results of Rv0986(NetMHCIIpan version 4.0)**

| Allele | start | end | peptide | Score | Percentile Rank |
| --- | --- | --- | --- | --- | --- |
| HLA-DRB1*07:01 | 198 | 212 | KTLIMATHSPSMTQH | 0.731840 | 0.60 |
| HLA-DRB1*07:01 | 197 | 211 | GKTLIMATHSPSMTQ | 0.691573 | 0.75 |
| HLA-DRB1*07:01 | 196 | 210 | AGKTLIMATHSPSMT | 0.570712 | 1.34 |
| HLA-DRB1*07:01 | 199 | 213 | TLIMATHSPSMTQHA | 0.390163 | 2.70 |
| HLA-DRB1*07:01 | 94 | 108 | FQFFNLIPTLTVLEN | 0.351662 | 3.14 |
| HLA-DRB1*07:01 | 195 | 209 | QAGKTLIMATHSPSM | 0.287807 | 4.08 |
| HLA-DRB1*07:01 | 93 | 107 | VFQFFNLIPTLTVLE | 0.265661 | 4.45 |
| HLA-DRB1*07:01 | 151 | 165 | EQQRVAISRALAHNP | 0.260133 | 4.56 |
| HLA-DRB1*07:01 | 150 | 164 | GEQQRVAISRALAHN | 0.243787 | 4.89 |
| HLA-DRB1*07:01 | 152 | 166 | QQRVAISRALAHNPM | 0.224638 | 5.35 |

| Allele | start | end | peptide | Score | Percentile Rank |
| --- | --- | --- | --- | --- | --- |
| HLA-DRB1*03:01 | 69 | 83 | INGFAITQKTERDRT | 0.547371 | 1.75 |
| HLA-DRB1*03:01 | 75 | 89 | TQKTERDRTLFRRDQ | 0.539788 | 1.79 |
| HLA-DRB1*03:01 | 68 | 82 | TINGFAITQKTERDR | 0.523919 | 1.88 |
| HLA-DRB1*03:01 | 74 | 88 | ITQKTERDRTLFRRD | 0.471336 | 2.24 |
| HLA-DRB1*03:01 | 185 | 199 | VLDVLLDLTRQAGKT | 0.447633 | 2.41 |
| HLA-DRB1*03:01 | 184 | 198 | KVLDVLLDLTRQAGK | 0.414751 | 2.68 |
| HLA-DRB1*03:01 | 70 | 84 | NGFAITQKTERDRTL | 0.397457 | 2.83 |
| HLA-DRB1*03:01 | 67 | 81 | VTINGFAITQKTERD | 0.375426 | 3.02 |
| HLA-DRB1*03:01 | 174 | 188 | TGNLDSDTGDKVLDV | 0.373255 | 3.04 |
| HLA-DRB1*03:01 | 173 | 187 | PTGNLDSDTGDKVLD | 0.345797 | 3.29 |

| Allele | start | end | peptide | Score | Percentile Rank |
| --- | --- | --- | --- | --- | --- |
| HLA-DRB1*15:01 | 100 | 114 | IPTLTVLENITLPQE | 0.664703 | 0.94 |
| HLA-DRB1*15:01 | 99 | 113 | LIPTLTVLENITLPQ | 0.553067 | 1.42 |
| HLA-DRB1*15:01 | 213 | 227 | ADRVVNLQGGRLIPA | 0.488279 | 1.75 |
| HLA-DRB1*15:01 | 101 | 115 | PTLTVLENITLPQEL | 0.451839 | 1.97 |
| HLA-DRB1*15:01 | 212 | 226 | HADRVVNLQGGRLIP | 0.391985 | 2.42 |
| HLA-DRB1*15:01 | 214 | 228 | DRVVNLQGGRLIPAV | 0.336756 | 2.91 |
| HLA-DRB1*15:01 | 98 | 112 | NLIPTLTVLENITLP | 0.316556 | 3.12 |
| HLA-DRB1*15:01 | 211 | 225 | QHADRVVNLQGGRLI | 0.182857 | 5.42 |
| HLA-DRB1*15:01 | 198 | 212 | KTLIMATHSPSMTQH | 0.166865 | 5.87 |
| HLA-DRB1*15:01 | 3 | 17 | RQPIVQLSNLSWTFR | 0.152837 | 6.31 |
