# Supplementary material for: Development of innovative multi-epitope mRNA vaccine against central nervous system tuberculosis using in silico approaches
Source: PLoS One. 2024 Sep 6;19(9):e0307877. doi: 10.1371/journal.pone.0307877 (PMC11379207; doi:10.1371/journal.pone.0307877)
Supplement: S7 Table — (DOCX) [file pone.0307877.s007.docx]

**PLOS ONE**

**Article title:Development of innovative multi-epitope mRNA vaccine against central nervous system tuberculosis using in silico approaches**

**Author:Huidong Shi**

**S7 Table. MHC-Ⅱ Binding Prediction Results of PknD(IEDB)**

| Allele | start | end | peptide | Score | Percentile Rank |
| --- | --- | --- | --- | --- | --- |
| HLA-DRB1*07:01 | 359 | 373 | PNALQASLGHAVPPA | 0.8913 | 0.19 |
| HLA-DRB1*07:01 | 358 | 372 | VPNALQASLGHAVPP | 0.8739 | 0.22 |
| HLA-DRB1*07:01 | 107 | 121 | YGPLTPARAVAIVRQ | 0.8578 | 0.28 |
| HLA-DRB1*07:01 | 106 | 120 | QYGPLTPARAVAIVR | 0.8293 | 0.36 |
| HLA-DRB1*07:01 | 421 | 435 | GIDFRLSPSGVAVDS | 0.7742 | 0.62 |
| HLA-DRB1*07:01 | 357 | 371 | AVPNALQASLGHAVP | 0.7605 | 0.72 |
| HLA-DRB1*07:01 | 420 | 434 | TGIDFRLSPSGVAVD | 0.7532 | 0.75 |
| HLA-DRB1*07:01 | 105 | 119 | KQYGPLTPARAVAIV | 0.7322 | 0.86 |
| HLA-DRB1*07:01 | 123 | 137 | AAALDAAHANGVTHR | 0.6852 | 1.10 |
| HLA-DRB1*07:01 | 108 | 122 | GPLTPARAVAIVRQI | 0.6912 | 1.10 |

| Allele | start | end | peptide | Score | Percentile Rank |
| --- | --- | --- | --- | --- | --- |
| HLA-DRB1*03:01 | 512 | 526 | PEGLAVDTQGAVYVA | 0.8868 | 0.39 |
| HLA-DRB1*03:01 | 596 | 610 | PWGIAVDEAGTVYVT | 0.8712 | 0.46 |
| HLA-DRB1*03:01 | 511 | 525 | YPEGLAVDTQGAVYV | 0.8451 | 0.58 |
| HLA-DRB1*03:01 | 595 | 609 | APWGIAVDEAGTVYV | 0.8440 | 0.59 |
| HLA-DRB1*03:01 | 428 | 442 | PSGVAVDSAGNVYVT | 0.8204 | 0.71 |
| HLA-DRB1*03:01 | 554 | 568 | PDGVAVDNSGNVYVT | 0.8187 | 0.72 |
| HLA-DRB1*03:01 | 183 | 197 | PERFTGDEVTYRADI | 0.8021 | 0.81 |
| HLA-DRB1*03:01 | 182 | 196 | APERFTGDEVTYRAD | 0.7995 | 0.84 |
| HLA-DRB1*03:01 | 427 | 441 | SPSGVAVDSAGNVYV | 0.7896 | 0.90 |
| HLA-DRB1*03:01 | 553 | 567 | DPDGVAVDNSGNVYV | 0.7857 | 0.93 |

| Allele | start | end | peptide | Score | Percentile Rank |
| --- | --- | --- | --- | --- | --- |
| HLA-DRB1*15:01 | 584 | 598 | NQVVLPFTDITAPWG | 0.8308 | 0.45 |
| HLA-DRB1*15:01 | 459 | 473 | GTTVLPFNGLYQPQG | 0.8305 | 0.46 |
| HLA-DRB1*15:01 | 626 | 640 | TSTVLPFTGLNTPLA | 0.8242 | 0.48 |
| HLA-DRB1*15:01 | 458 | 472 | TGTTVLPFNGLYQPQ | 0.7685 | 0.65 |
| HLA-DRB1*15:01 | 481 | 495 | TVYVTDFNNRVVTLA | 0.7491 | 0.70 |
| HLA-DRB1*15:01 | 625 | 639 | TTSTVLPFTGLNTPL | 0.7495 | 0.70 |
| HLA-DRB1*15:01 | 583 | 597 | NNQVVLPFTDITAPW | 0.7429 | 0.74 |
| HLA-DRB1*15:01 | 500 | 514 | NQTVLPFDGLNYPEG | 0.7400 | 0.75 |
| HLA-DRB1*15:01 | 624 | 638 | STTSTVLPFTGLNTP | 0.6822 | 1.10 |
| HLA-DRB1*15:01 | 480 | 494 | GTVYVTDFNNRVVTL | 0.6515 | 1.20 |
