# Supplementary material for: Development of innovative multi-epitope mRNA vaccine against central nervous system tuberculosis using in silico approaches
Source: PLoS One. 2024 Sep 6;19(9):e0307877. doi: 10.1371/journal.pone.0307877 (PMC11379207; doi:10.1371/journal.pone.0307877)
Supplement: S8 Table — (DOCX) [file pone.0307877.s008.docx]

**PLOS ONE**

**Article title:Development of innovative multi-epitope mRNA vaccine against central nervous system tuberculosis using in silico approaches**

**Author:Huidong Shi**

**S8 Table. MHC-Ⅱ Binding Prediction Results of PknD(NetMHCIIpan version 4.0)**

| Allele | start | end | peptide | Score | Percentile Rank |
| --- | --- | --- | --- | --- | --- |
| HLA-DRB1*07:01 | 107 | 121 | YGPLTPARAVAIVRQ | 0.836374 | 0.31 |
| HLA-DRB1*07:01 | 359 | 373 | PNALQASLGHAVPPA | 0.812997 | 0.37 |
| HLA-DRB1*07:01 | 106 | 120 | QYGPLTPARAVAIVR | 0.808413 | 0.38 |
| HLA-DRB1*07:01 | 358 | 372 | VPNALQASLGHAVPP | 0.788851 | 0.43 |
| HLA-DRB1*07:01 | 421 | 435 | GIDFRLSPSGVAVDS | 0.683527 | 0.78 |
| HLA-DRB1*07:01 | 105 | 119 | KQYGPLTPARAVAIV | 0.681266 | 0.79 |
| HLA-DRB1*07:01 | 123 | 137 | AAALDAAHANGVTHR | 0.671201 | 0.83 |
| HLA-DRB1*07:01 | 108 | 122 | GPLTPARAVAIVRQI | 0.663150 | 0.86 |
| HLA-DRB1*07:01 | 420 | 434 | TGIDFRLSPSGVAVD | 0.660375 | 0.87 |
| HLA-DRB1*07:01 | 357 | 371 | AVPNALQASLGHAVP | 0.659046 | 0.87 |

| Allele | start | end | peptide | Score | Percentile Rank |
| --- | --- | --- | --- | --- | --- |
| HLA-DRB1*03:01 | 512 | 526 | PEGLAVDTQGAVYVA | 0.892460 | 0.28 |
| HLA-DRB1*03:01 | 511 | 525 | YPEGLAVDTQGAVYV | 0.866764 | 0.36 |
| HLA-DRB1*03:01 | 182 | 196 | APERFTGDEVTYRAD | 0.859672 | 0.38 |
| HLA-DRB1*03:01 | 183 | 197 | PERFTGDEVTYRADI | 0.857761 | 0.39 |
| HLA-DRB1*03:01 | 596 | 610 | PWGIAVDEAGTVYVT | 0.830180 | 0.47 |
| HLA-DRB1*03:01 | 640 | 654 | AVAVDSDRTVYVADR | 0.814489 | 0.52 |
| HLA-DRB1*03:01 | 595 | 609 | APWGIAVDEAGTVYV | 0.807140 | 0.55 |
| HLA-DRB1*03:01 | 510 | 524 | NYPEGLAVDTQGAVY | 0.802603 | 0.57 |
| HLA-DRB1*03:01 | 554 | 568 | PDGVAVDNSGNVYVT | 0.786806 | 0.63 |
| HLA-DRB1*03:01 | 563 | 577 | GNVYVTDTDNNRVVK | 0.778710 | 0.66 |

| Allele | start | end | peptide | Score | Percentile Rank |
| --- | --- | --- | --- | --- | --- |
| HLA-DRB1*15:01 | 584 | 598 | NQVVLPFTDITAPWG | 0.831549 | 0.43 |
| HLA-DRB1*15:01 | 459 | 473 | GTTVLPFNGLYQPQG | 0.762121 | 0.62 |
| HLA-DRB1*15:01 | 626 | 640 | TSTVLPFTGLNTPLA | 0.759244 | 0.63 |
| HLA-DRB1*15:01 | 583 | 597 | NNQVVLPFTDITAPW | 0.752196 | 0.65 |
| HLA-DRB1*15:01 | 481 | 495 | TVYVTDFNNRVVTLA | 0.713219 | 0.77 |
| HLA-DRB1*15:01 | 458 | 472 | TGTTVLPFNGLYQPQ | 0.691907 | 0.83 |
| HLA-DRB1*15:01 | 390 | 404 | LVAIVAAAGYLVLRP | 0.682664 | 0.87 |
| HLA-DRB1*15:01 | 625 | 639 | TTSTVLPFTGLNTPL | 0.672742 | 0.91 |
| HLA-DRB1*15:01 | 582 | 596 | SNNQVVLPFTDITAP | 0.667889 | 0.93 |
| HLA-DRB1*15:01 | 480 | 492 | GTVYVTDFNNRVVTL | 0.629290 | 1.08 |
