# Supplementary material for: Development of innovative multi-epitope mRNA vaccine against central nervous system tuberculosis using in silico approaches
Source: PLoS One. 2024 Sep 6;19(9):e0307877. doi: 10.1371/journal.pone.0307877 (PMC11379207; doi:10.1371/journal.pone.0307877)
Supplement: S9 Table — (DOCX) [file pone.0307877.s009.docx]

**PLOS ONE**

**Article title:Development of innovative multi-epitope mRNA vaccine against central nervous system tuberculosis using in silico approaches**

**Author:Huidong Shi**

**S9 Table. LBEs Results of Rv0986 and PknD(SVMtrip)**

|  | start | end | peptide | Rank | Score |
| --- | --- | --- | --- | --- | --- |
| Rv0986 | 100 | 119 | IPTLTVLENITLPQELAGVS | 1 | 1.000 |
|  | 192 | 211 | LTRQAGKTLIMATHSPSMTQ | 2 | 0.730 |
|  | 61 | 80 | KPTTGDVTINGFAITQKTER | 3 | 0.562 |
|  | 152 | 171 | QQRVAISRALAHNPMLVLAD | 4 | 0.523 |
| PknD | 50 | 69 | YSDNAVFRARMQREADTAGR | 1 | 1.000 |
|  | 87 | 106 | QFFVEMRMIDGTSLRALLKQ | 2 | 0.914 |
|  | 107 | 126 | YGPLTPARAVAIVRQIAAAL | 3 | 0.870 |
|  | 288 | 307 | LRRGDNATLLATPADTGLSQ | 4 | 0.567 |
|  | 243 | 262 | PPALDQVIAKGMAKNPAERF | 5 | 0.555 |
|  | 572 | 591 | NNRVVKLEAESNNQVVLPFT | 6 | 0.551 |
