# Supplementary figures and images for: Development of innovative multi-epitope mRNA vaccine against central nervous system tuberculosis using in silico approaches
Source: PLoS One. 2024 Sep 6;19(9):e0307877. doi: 10.1371/journal.pone.0307877 (PMC11379207; doi:10.1371/journal.pone.0307877)

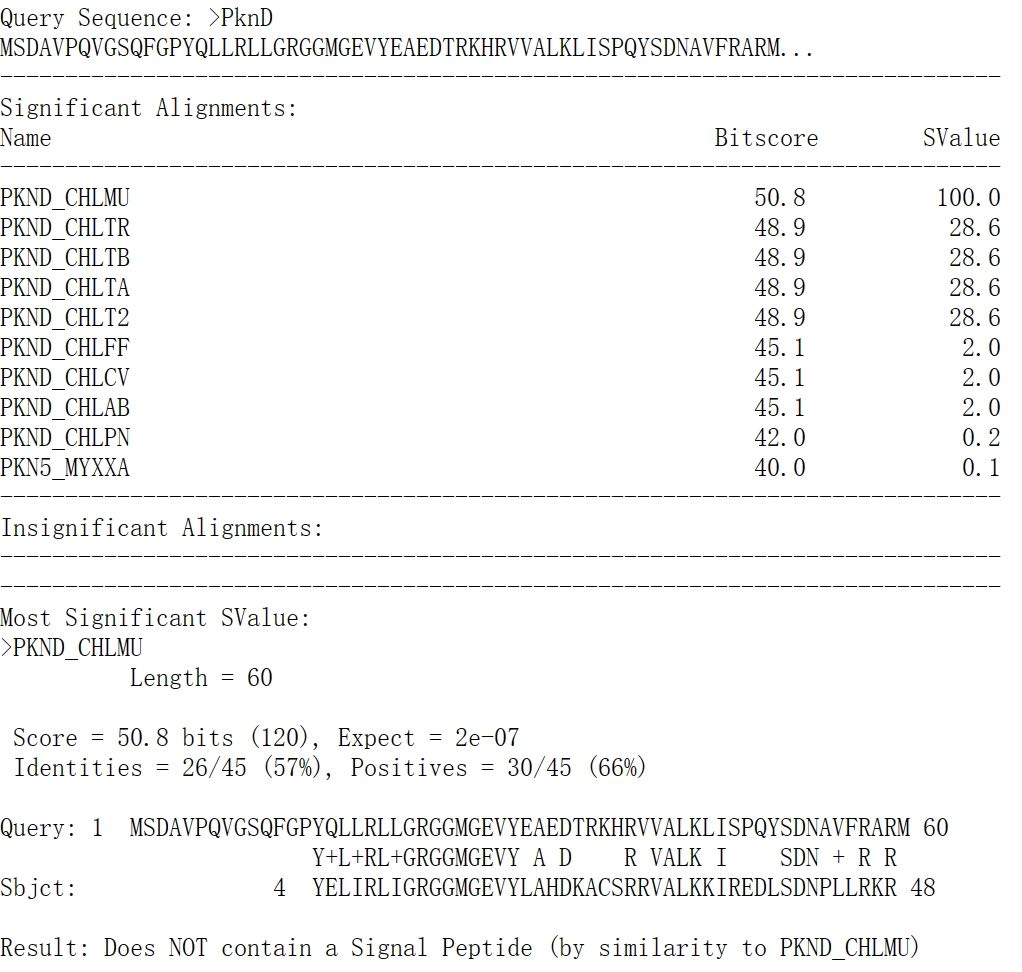

Supplement: S1 Fig — (TIF) [file pone.0307877.s010.tif]

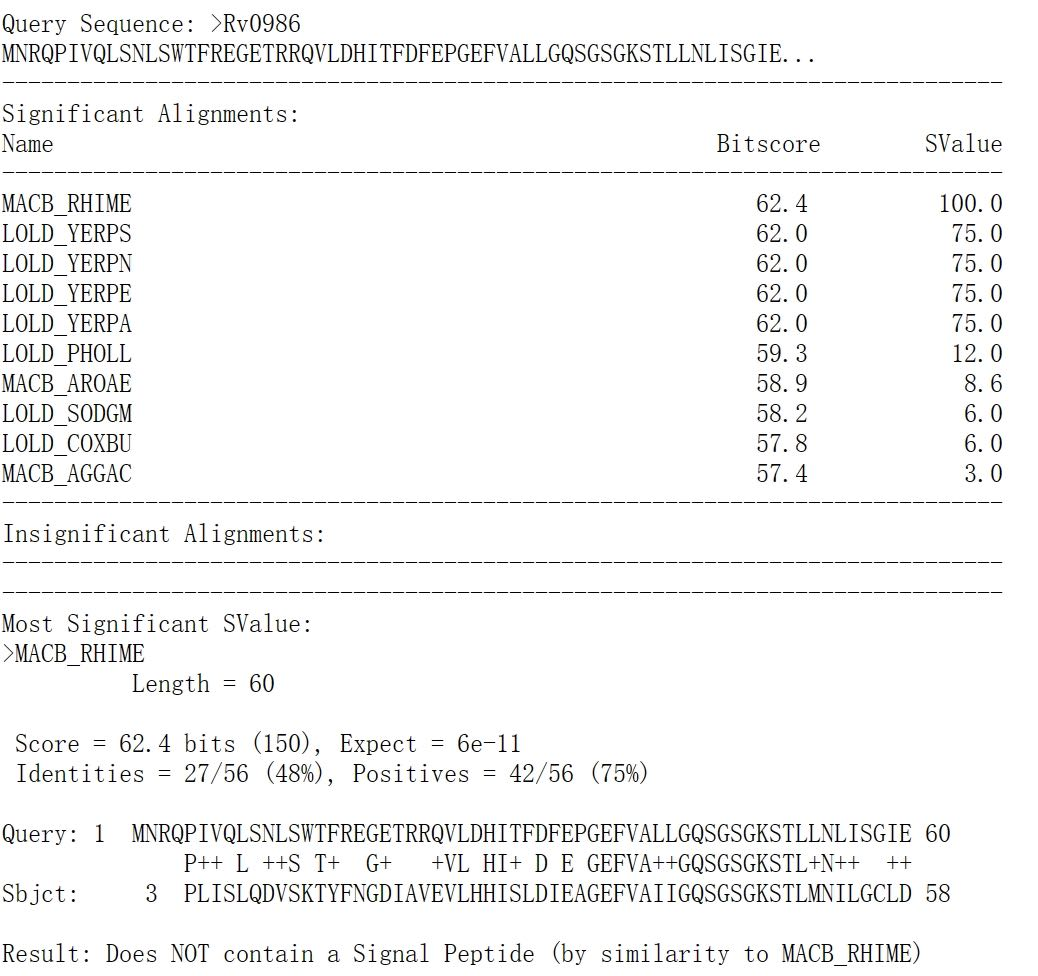

Supplement: S2 Fig — (TIF) [file pone.0307877.s011.tif]

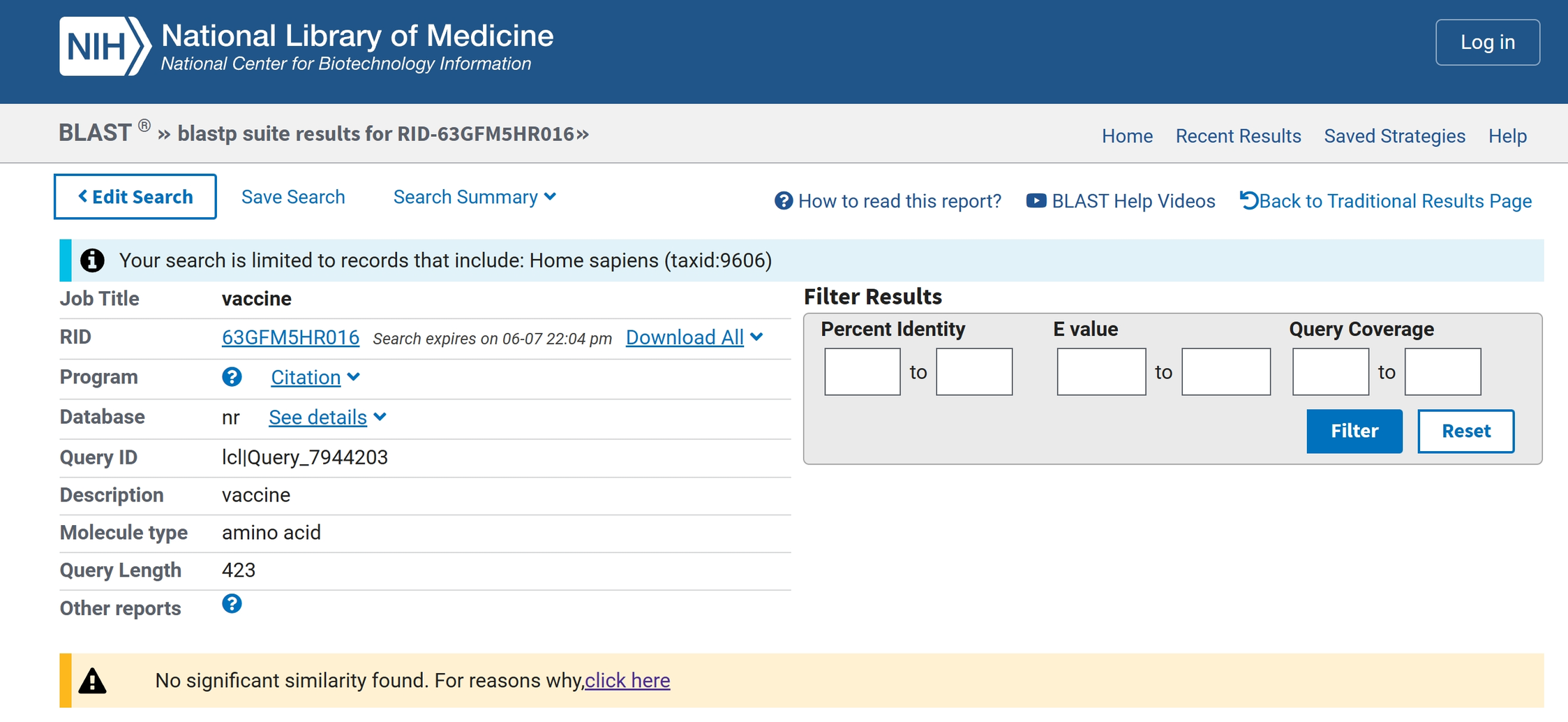

Supplement: S3 Fig — (TIF) [file pone.0307877.s012.tif]
