# Supplementary material for: Development of innovative multi-epitope mRNA vaccine against central nervous system tuberculosis using in silico approaches
Source: PLoS One. 2024 Sep 6;19(9):e0307877. doi: 10.1371/journal.pone.0307877 (PMC11379207; doi:10.1371/journal.pone.0307877)
Supplement: Attachment — Submitted filename: Comments_PONE-D-24-15467.docx [file pone.0307877.s014.docx]

1. **Overview and general recommendation:**

Reading the paper, I found the overall language easy to read, covering a lot of information through description of the various techniques applied & discussion of results, and relevant literature included.

2. **Major Revisions**
   2.1 What is the rationale for selecting the epitome length. Why epitome length was set to 15 and 20 for T cell and B cell epitopes respectively.
   2.2 Line 277, kindly clarify which linker has what properties and accordingly change the line. Or all three linkers are rigid as well as flexible?
   2.3 Section 3.8, Author has evaluated physicochemical properties however, antigenicity, sensitization and toxicity is missing. Kindly provide the results by which you claim the antigenicity, sensitization and toxicity of MEV
3. **Minor Revisions**
   3.1 In Introduction line 74, 75 abbreviations are used like IEDB, CTL, HTL, However, write full form for the first time along with the abbreviations. Later you may use only abbreviations.
   3.2 In Line 88, write the version of MAFFT software.
   3.3 Write the software name with its version elsewhere applicable in manuscript.
   3.4 Table 4 missing the title raw.
   3.5 Write the full form of BES.
