# Supplementary material for: Development of innovative multi-epitope mRNA vaccine against central nervous system tuberculosis using in silico approaches
Source: PLoS One. 2024 Sep 6;19(9):e0307877. doi: 10.1371/journal.pone.0307877 (PMC11379207; doi:10.1371/journal.pone.0307877)
Supplement: Attachment — Submitted filename: Response to Reviewers.docx [file pone.0307877.s016.docx]

Dear editor & reviewers,

Thank you for your kind patience. We have revised our manuscript (Submission ID: PONE-D-24-15467) carefully by following the guidance provided by the editor and reviewers. Here are the point-by-point responses for the editor and reviewers’ comments.

**Response to Academic Editor**

1.Please ensure that your manuscript meets PLOS ONE's style requirements.

Response:Dear academic editor,thank you for reviewing our research article and providing valuable suggestions for revisions.We have changed the style of the literature according to the requirements of the PLOS ONE journal reference.

2. Please note that PLOS ONE has specific guidelines on code sharing for submissions in which author-generated code underpins the findings in the manuscript. In these cases, we expect all author-generated code to be made available without restrictions upon publication of the work.

Response:Dear academic editor,thank you for reviewing our research article and providing valuable suggestions for revisions. We are very willing to share the code, We have stored laboratory program in a separate protocols.io,generate DOI: dx.doi.org/10.17504/protocols.io.81wgbzr31gpk/v1.; The generated link is: https://www.protocols.io/private/C80855412E6C11EFA80E0A58A9FEAC02, is available for editors and referees to check. We added this section to the Data availability statement. If you need any information from us, please feel free to communicate with us.

3. Please upload a new copy of Figures 1, 2, 3A, 3B, 6, 7, 8, 9A, 9B, 9C, 12A and 14 as the detail is not clear.

Response:Dear academic editor,thank you for reviewing our research article and providing valuable suggestions for revisions.For ease of understanding, we have reuploaded all images as per the magazine's image requirements.

**Response to Reviewer1**

This insilico approach of developing a multiepitope vaccine for CNS TB is an innovative and thoroughly carried out study. The comprehensive bioinformatics analyses, including sequence homology, antigenicity prediction, and epitope mapping, contribute to a comprehensive understanding of the vaccine candidates' immunogenic potential. Additionally, the incorporation of immunoinformatic tools facilitates the prediction of T-cell and B-cell epitopes, further enhancing the vaccine's efficacy and specificity. Despite the remarkable accomplishments of this study, I have few questions and minor concerns that needs to be addressed.

Response:Dear reviewer,thank you for reviewing our research article and providing valuable suggestions for revisions. We take your feedback seriously and have made corresponding revisions based on your suggestions. Here is our response to your proposed modification suggestions:

1.Line 229: Claim “the two proteins are highly homologous, suggesting that they may be derived from the same gene and have similar roles in the immune response”. Did you find any link between the two genes in the previous literatures or perform any additional study to investigate the same other than sequence homology?

Response:Dear reviewer, thank you for your valuable suggestions.In previous studies, pknD and Rv0986 genes have been shown to be involved in invasion of the central nervous system and are specifically expressed in the central nervous system, missing in lung tissue. In the Uniprot database, both genes were found to be from the H37Rv strain. The references are as follows：

(1).Be NA, Bishai WR, Jain SK. Role of Mycobacterium tuberculosis pknD in the pathogenesis of central nervous system tuberculosis. *BMC Microbiol*. 2012;12:7. Published 2012 Jan 13. doi:10.1186/1471-2180-12-7；

(2).Be NA, Lamichhane G, Grosset J, et al. Murine model to study the invasion and survival of Mycobacterium tuberculosis in the central nervous system [published correction appears in J Infect Dis. 2009 Jan 15;199(2):290]. *J Infect Dis*. 2008;198(10):1520-1528. doi:10.1086/592447；

(3).Jain SK, Paul-Satyaseela M, Lamichhane G, Kim KS, Bishai WR. Mycobacterium tuberculosis invasion and traversal across an in vitro human blood-brain barrier as a pathogenic mechanism for central nervous system tuberculosis. *J Infect Dis*. 2006;193(9):1287-1295. doi:10.1086/502631.

These articles are cited in manuscripts.

2.Did you check similarity between analyzed bacterial proteins and host proteome?

Response:Dear reviewer, thank you for your valuable suggestions.We build a multi-epitope vaccine, and the sequence used to build the vaccine is just a part of the protein. In order to avoid homology between the vaccine and the host, we used the BlastP database to compare homology between the vaccine and Homo sapiens (TaxID: 9606). An E value greater than 0.5 is considered non-homologous and suitable for vaccine construction. The results showed that no significant correlation was found between the two. We have added this section in Line145-149 and Line 324-326.The screenshot of the result is in the supporting information and is called S3(TIF).

3.Did you by any chance used another software such as Signal BLAST to predict signal peptide?

Response:Dear reviewer, thank you for your valuable suggestions.According to your suggestion, we used Signal BLAST to predict the signal peptides of the proteins PknD and Rv0986 again, and the results showed that there were no signal peptides here.The screenshot of the result is in the supporting information and is called S1(TIF), S2 (TIF).

4. Would you have any other already tested CTL and HTL epitope from previous CNS TB studies / published literature which can be used as comparison with your selected epitope candidates to validate how selected epitope candidates stand out or performing better.

Response:Dear reviewer, thank you for your valuable suggestions.The proteins we selected appear specifically in the central nervous system, and there is currently no literature analyzing multiepitope vaccines for CNS TB, which is also the innovation of our paper. However, there are many literature reports on tuberculosis multi-epitope vaccines. Compared with their selected CTL/HTL epitopes, the epitopes we selected all have higher antigenicity, non-allergenic, non-toxic, and good physicochemical properties. The references are as follows:

(1).Andongma BT, Huang Y, Chen F, et al. *In silico* design of a promiscuous chimeric multi-epitope vaccine against *Mycobacterium tuberculosis*. *Comput Struct Biotechnol J*. 2023;21:991-1004. Published 2023 Jan 16. doi:10.1016/j.csbj.2023.01.019

(2).Al Tbeishat H. Novel In Silico mRNA vaccine design exploiting proteins of M. tuberculosis that modulates host immune responses by inducing epigenetic modifications. *Sci Rep*. 2022;12(1):4645. Published 2022 Mar 17. doi:10.1038/s41598-022-08506-4

(3).Jiang F, Han Y, Liu Y, et al. A comprehensive approach to developing a multi-epitope vaccine against *Mycobacterium tuberculosis*: from *in silico* design to *in vitro* immunization evaluation. *Front Immunol*. 2023;14:1280299. Published 2023 Nov 2. doi:10.3389/fimmu.2023.1280299

Major concern

1: Most of the figures are hard to read and see. Image quality is poor and written text is unclear. Please replace with good quality images.

Response:Dear reviewer, thank you for your valuable suggestions.We have made changes to the quality and resolution of all images in accordance with the magazine's image requirements.

Minor concern:

1. There are various formatting errors like invalid spacing between words in headings and paragraphs. Please check thoroughly.

Response:Dear reviewer, thank you for your valuable suggestions.We have thoroughly checked the word spacing between the words in headings and paragraph in the manuscript to ensure compliance with the journal requirements.

2. Line 40: Rephrase. Why would TB report 2022 will predict cases in 2021. Prediction happens for future.

Response:Dear reviewer,we are sorry for the tense error due to our negligence. We have rephrase the sentence in line 39-40.

3.Line 260: “Docking result between CTLs and HLA-A*02:01 seems confusing. Please rephrase this. Does this mean CTL epitope and HLA-A*02:01?

Response:Dear reviewer,We are very sorry for the error of expression due to our carelessness. In line 260, we want to express the choice of an ITAPWGIAV epitope in the CTLs to interface with HLA-A*02:01. We have corrected it in the line 302.

4. Line 261: Similarly, “Docking result between HTLs and HLA-DRB1*07:01” seems confusing.

Response:We are very sorry for the error of expression due to our carelessness. In line 261, we want to express that one of the FQFFNLIPTLTVLEN opes in HTLs is selected to interface with HLA-DRB1*07:01. We have corrected it in the line 303-304.

5.Section 3.8: Please make a table for MEV properties instead of writing the properties in sentences.

Response:Dear reviewer, thank you for your valuable suggestions.We have made a table for MEV properties and named it Table 6.

6.Section 3.9: Would it be possible to extrapolate dose and time intervals of injection during simulation of immune response?

Response:Dear reviewer,we have described in section 2.10 three doses of the vaccine for 1 day, 84 days, 168 days, each dose of 1000 units.Based on the simulations, the doses and intervals we injected were reasonable. We have added this sentence to line 349.

7.3.12 and 3.13 heading both has prediction of secondary structure. Why?

Response:Dear reviewer, 3.12 predicts the secondary structure of mRNA in order to ensure the effectiveness of mRNA transcription. 3.13 predicts the secondary structure of the vaccine protein in order to ensure the validity of the translation. For ease of understanding, we have been explained in the 2.12 and 2.13.

8.Table 4 does not have heading for characteristics measured. No row to define which column represents which characteristics. Include antigenicity values in table 5.

Response:Dear reviewer, we are sorry for the problem caused by our oversight. We have added a title to Table4. The antigenicity value is added in Table5. Finally, the antigenicity of MEV we constructed is 0.9510, which has strong antigenicity.

**Response to Reviewer2**

1.Please use a correct font, font size and text settings for the manuscript

Response:Dear reviewer, thank you for your valuable suggestions.We have used the correct font and font size for the text in the manuscript as required by the journal. Strains and restriction enzyme names are used in italics.

2.Define all the abbreviations in the manuscript for the words where they are used for the first time.

Response:Dear reviewer, thank you for your valuable suggestions.We have defined all the abbreviations used for the first time in the manuscript.

3.Please use the correct style for the citations in the manuscript.

Response:Dear reviewer, thank you for your valuable suggestions.We have changed the style of the literature according to the requirements of the PLOS ONE journal reference.

4. The quality and resolution of the images needs to be improved, the aspect ratio for some of the images is incorrect.

Response:Dear reviewer, thank you for your valuable suggestions.We have made changes to the quality and resolution of all images in accordance with the magazine's image requirements.

5. Please remove the characters from the other languages from the manuscript.

Response:Dear reviewer, thank you for your valuable suggestions.We have checked the language and font in the manuscript to make sure it meets the requirements of the journal.

6.please try to reduce the number of images from manuscript, possibly by merging them together.

Response:Dear reviewer, thank you for your valuable suggestions.We have merged Fig3A,Fig3B into Fig3, Fig9A,B,C into Fig9, Fig11A,B into Fig11, Fig12A,B,C,D into Fig12, Fig13A,B,C into Fig13, Fig14A,B,C into Fig14.

**Response to Reviewer3**

General comments:

The authors of this study aimed to develop an innovative mRNA therapeutic vaccine targeting a protein associated with central nervous system tuberculosis, addressing the pressing need for effective treatments for this debilitating condition. Employing in silico techniques such as IEDB, NetCTLpan1.1, NetMHCIIpan-4.0, and SVMtrip, they analyzed the antigen epitopes of PknD and Rv0986. Additionally, CTL, HTL, and B cell epitopes were linked together using AAY, GPGPG, and KK linkers. This research was spurred by the lack of therapeutic options for central nervous system tuberculosis, underscoring its urgency.

The utilization of in silico methods paved the way for designing an mRNA therapeutic vaccine targeting central nervous system tuberculosis, offering a foundation for potential future experimental investigations. The outcomes of these assessments yielded promising observations, indicating the potential efficacy of the developed vaccine.

The authors have presented promising results in the manuscript. However, to optimize its effectiveness for our readers, it would be beneficial to address the following comments for streamlining and enhancement. While the manuscript exhibits good writing and organization, improvements in overall English language usage, particularly in grammar, punctuation, and clarity, would enhance engagement for general readers. Please consider revising the language to maximize effectiveness

Response:Dear reviewer,thank you for your full recognition of our manuscript, we have fully considered your comments, made grammatical changes to the full text, checked the use of punctuation, and re-uploaded all images in accordance with the magazine's requirements for image clarity. Here is our response to your comments.

1. Line 26: Define ‘BCG’ or provide more descriptive information as it appears first time here in this manuscript. Applicable for all abbreviations throughout the manuscript.

Response:Dear reviewer, thank you for your valuable suggestions.We have defined BCG and added it to the abbreviations at the end of the manuscript .

2. Line 33-34: please revise this sentence for effectiveness: ‘The results indicate that the vaccine structure shows promise’.

Response:Dear reviewer, thank you for your valuable suggestions.We have rephrased the sentence in lines 32.

3.Line 40-41: The tense of the following sentence needs to be revised. If updated and most recent data is available, please provide that. ‘The World Health Organization (WHO) Global TB Report 2022 predicts that 41 10.6 million people will be diagnosed with TB in 2021, with 1.6 million fatalities.

Response:Dear reviewer, we are sorry for the tense error due to our negligence. We have rephrase the sentence in line 39-40.

4.Line 42: Consider defining as this term is used many times in the manuscript ‘Central nervous system tuberculosis’.

Response:Dear reviewer, thank you for your valuable suggestions.We have defined central nervous system tuberculosis in line 23.

5.Line 42: ‘TB’ is not defined, please define it.

Response:Dear reviewer, thank you for your valuable suggestions.We have defined TB in line 23.

6.Line 44: If authors could provide specifics in terms of specific mortality rate from the cited article, that would be helpful for readers.

Response:Dear reviewer, thank you for your valuable suggestions.We have provided specific details of mortality in line 42-44 and have cited relevant literature.The references are as follows:

(1).Wilkinson RJ, Rohlwink U, Misra UK, et al. Tuberculous meningitis. *Nat Rev Neurol*. 2017;13(10):581-598. doi:10.1038/nrneurol.2017.120

(2).Mezochow A, Thakur K, Vinnard C. Tuberculous Meningitis in Children and Adults: New Insights for an Ancient Foe. *Curr Neurol Neurosci Rep*. 2017;17(11):85. Published 2017 Sep 20. doi:10.1007/s11910-017-0796-0

7.Line 77 to 83: This paragraph has three references cited. This is confusing. Have these previous studies been conducted already? if they are, how they are related to the current study is not clear? please revise the language for clarity. If the intention is to cite for the part of the study, please make it clear.

Response:Dear reviewer, thank you for your valuable suggestions.In lines 77-83, we only want to refer to the method part of the literature. The literature is relevant to the design of multi-epitope vaccines, so these methods are also applicable in this study.We have explained this in lines 73-74

8. Please revise the whole manuscript for formatting, such as: provide space between the last word in the sentence and the reference parenthesis. Applicable throughout the manuscript.

Response:Dear reviewer, thank you for your valuable suggestions.We have followed your suggestion to leave a space between the last word of the sentence and the reference brackets.

9. Line 87: link for the database should be before full stop. Please check the formatting throughout the manuscript.

Response:Dear reviewer, thank you for your valuable suggestions.We have followed your suggestion to place the database link before the period and apply it in the full manuscript.

10.1All figures: Presently, all figures appear distorted and oddly stretched. Please recreate them with appropriate resolution and dimensions to ensure clarity. Additionally, consider increasing the font size on the figures for improved legibility. Some content on certain figures is currently illegible due to distortion. This feedback applies to all figures.

Response:Dear reviewer, thank you for your valuable suggestions.We have made changes to the quality and resolution of all images in accordance with the magazine's image requirements.At the same time, the number size on the image is increased, and the readability is improved.

11. All section titles: provide a space between the number and section title, applicable for all section titles.

Response:Dear reviewer, thank you for your valuable suggestions.We have followed your suggestion to leave Spaces between all section titles and numbers.

12. Validation of Models: It is advisable to elaborate on the validation process of the models utilized in this study to assess their reliability. Please address any limitations associated with validating these models to provide insight for readers or researchers interested in utilizing them in the future.

Response:Dear reviewer, thank you for your valuable suggestions.We have added the model validation process in the line 206-224,399-405 and explained the model limitations, while citing references (50)(51). The amino acid residues in the optimal region of the model we constructed accounted for 92.1% of the whole protein, which was greater than 90%. Amino acid residues in the disallowed region account for 1.4% of the total protein, less than 5%. The energy diagram shows that most of the sequence positions are negative.All these prove that our model is reasonable.The details analytical procedure are described in manuscript 2.14.The references are as follows:

(1).Hooft RW, Sander C, Vriend G. Objectively judging the quality of a protein structure from a Ramachandran plot. *Comput Appl Biosci*. 1997;13(4):425-430. doi:10.1093/bioinformatics/13.4.425

(2).Wiederstein M, Sippl MJ. ProSA-web: interactive web service for the recognition of errors in three-dimensional structures of proteins. *Nucleic Acids Res*. 2007;35(Web Server issue):W407-W410. doi:10.1093/nar/gkm290

**Response to Reviewer4**

**1.Overview and general recommendation:**

Reading the paper, I found the overall language easy to read, covering a lot of information through description of the various techniques applied & discussion of results, and relevant literature included.

Response:Dear reviewer,thank you very much for your valuable comments on our article, the following are our amendments.

**2.Major Revisions**

2.1 What is the rationale for selecting the epitome length. Why epitome length was set to 15 and 20 for T cell and B cell epitopes respectively.

Response:Dear reviewer, thank you for your valuable suggestions.In order to construct multi-epitope vaccine, we selected T cell epitope and B cell epitope respectively, and finally connected with linker. The length of the vaccine will affect its expression, so an appropriate epitope length is necessary. These lengths are also used in other articles. Epitope length is too short to predict its physical and chemical properties, antigenicity, sensitization and toxicity. 15 and 20 are also the lengths recommended by the epitope prediction server NetMHCIIpan-4, SVMtrip.

2.2 Line 277, kindly clarify which linker has what properties and accordingly change the line. Or all three linkers are rigid as well as flexible?

Response:Dear reviewer, thank you for your valuable suggestions.We have rewritten that the linker AAY is breakable, GPGPG is rigid, and KK is flexible in line 323-324.

2.3 Section 3.8, Author has evaluated physicochemical properties however, antigenicity, sensitization and toxicity is missing. Kindly provide the results by which you claim the antigenicity, sensitization and toxicity of MEV

Response:Dear reviewer, thank you for your valuable suggestions.We have made Table 6, which includes antigenicity, sensitization and toxicity of MEV.The antigenicity of the MEV we constructed is 0.9510, with high antigenicity, non-sensitization and non-toxicity.

**3.Minor Revisions**

3.1 In Introduction line 74, 75 abbreviations are used like IEDB, CTL, HTL, However, write full form for the first time along with the abbreviations. Later you may use only abbreviations.

Response:Dear reviewer, thank you for your valuable suggestions.We have added the full forms of IEDB, CTL,HTL for the first time, later only use abbreviations.

3.2 In Line 88, write the version of MAFFT software.

Response:Dear reviewer, thank you for your valuable suggestions.We have added the MAFFT version in line 87.

3.3 Write the software name with its version elsewhere applicable in manuscript.

Response:Dear reviewer, thank you for your valuable suggestions.We have described the software versions that can be retrieved.

3.4 Table 4 missing the title raw.

Response:Dear reviewer,we are sorry that we forgot to add the title of Table4 due to our negligence, and we have modified it in the manuscript.

3.5 Write the full form of BES.

Response:Dear reviewer, thank you for your valuable suggestions.The complete form of BES is the B-cell epitopes, which includes LBEs and CBEs. We have marked it in line 480.

**Response to Reviewer5**

In this review, Shi et al, explained a in silico approach for the treatment of CNS Tuberculosis. The authors have explained a detail approach from the selection of the target sequences to codon optimization probabilities to ensure it’s translation in Human host. The authors needs to address these concerns

Response:Dear reviewer,thank you very much for your recognition of our manuscript, here are our responses to your questions

Major:

1.All the figures are not clear/ visible to understand the results.

Response:Dear reviewer, we are very sorry that this problem has been caused by our negligence.We have made changes to the quality and resolution of all images in accordance with the magazine image requirements.

2.Authors need to mention or provide the details of the length of the amino acids for each prediction like PknD (lenth:--a.a), Rv0986 (length :..a.a) which will be easily understandable for the reader when they are discussing about the signal peptide sequence or the % homologous sequences.

Response:Dear reviewer, thank you for your valuable suggestions.After our prediction, the length of PknD is 664aa and the length of Rv0986 is 248aa.We have added in line 254.

3.Figure 1: Schematic Representation of the flow of the work is not clear like after prediction of the signal peptide the authors are try to find the epitope sequences, it’s a continuous process but the authors mentioned as in prediction of signal peptide there are two different kind of stages like epitopes which is not correct. And in the later stages also. Please correct it.

Response:Dear reviewer, thank you for your valuable suggestions.Signaling peptides play a guiding, secretory role, directing our proteins into other suborganelles and even secreting them outside the cell. Signaling peptides are not involved in the composition of vaccines. Therefore, before selecting a cell epitope, we need to make sure that the signal peptide is not present in the amino acid sequence, which is a two-step process. As predicted, the protein we selected does not have signaling peptides. We explain this in draft 2.3 and 3.3. In addition, in has been reported in the literature, the signal peptide prediction and cell epitope choice is divided into two steps, the following is the references:

(1).Ru Z, Yu M, Zhu Y, et al. Immmunoinformatics-based design of a multi-epitope vaccine with CTLA-4 extracellular domain to combat Helicobacter pylori. *FASEB J*. 2022;36(4):e22252. doi:10.1096/fj.202101538RR

(2).Chen Z, Zhu Y, Sha T, et al. Design of a new multi-epitope vaccine against *Brucella* based on T and B cell epitopes using bioinformatics methods. *Epidemiol Infect*. 2021;149:e136. Published 2021 May 25. doi:10.1017/S0950268821001229

4.Figure 2: MAFFT in Jalview is not clear enough to analyze.

Response:Dear reviewer, thank you for your valuable suggestions.We have resubmitted the image as required by the magazine

5.In Lane 222: Authors mentioned the two proteins are highly homologous….What is the percentage of Homogeneity?. Please mention it.

Response:Dear reviewer, thank you for your valuable suggestions. In the process of vaccine construction, we pay more attention to whether the constructed multi-epitope mRNA vaccine has homology with the host. We have added this section in 2.8. The results showed that the vaccine had no homology with the human host. Here we can only determine the homology of two proteins based on the color shade. For ease of understanding, we have reworked Fig2 and reinterpreted it in lines 263-267.

6.In lane 231: Black parts means black bars or the black stretch or both which indicates the highly conserved amino acids. How many A.A/ how much percentage of the a.a are conserved. Please Mention it.

Response:Dear reviewer, thank you for your valuable suggestions.We have reworked Fig2. We judge the conservatism by the depth of the color. In the figure, blue represents the conservative sequence, and the darker the blue, the more conservative the sequence.

7.All the Figures are not clear so it’s very difficult to interpret the conclusion from the figures. (Example: Figure 8, nothing is visible to analyze the data).

Response:Dear reviewer, thank you for your valuable suggestions.We have resubmitted all images including Image 8, which meet the requirements of the magazine, with high resolution and high quality.

8.Signal Sequence: Lane 130: tPA signal aids in secreting the signal sequence of epitope. In the results section authors mentioned there is no signal sequence detected in both the proteins. Please correct it,

Response:Dear reviewer, thank you for your valuable suggestions.In Result 3.3, we predict the signal peptide of the selected protein, and our aim is to eliminate the signal peptide here, because it will guide the protein to other suborganelles of the cell, and also enable the protein to function outside the cell after synthesis. It is not included in vaccine synthesis. In Result 3.7, when we construct mRNA vaccines, tPA signaling peptide is a part of the vaccine structure, its main role is to ensure the correct translation of the target gene fragment, while enhancing the immunogenicity of the vaccine. This is advantageous. We've rewritten line 138. These effects have been reported in the literature, the references are as follows：

(1).Shi J, Zhu Y, Yin Z, et al. In silico designed novel multi-epitope mRNA vaccines against Brucella by targeting extracellular protein BtuB and LptD. *Sci Rep*. 2024;14(1):7278. Published 2024 Mar 27. doi:10.1038/s41598-024-57793-6

(2).Al Tbeishat H. Novel In Silico mRNA vaccine design exploiting proteins of M. tuberculosis that modulates host immune responses by inducing epigenetic modifications. *Sci Rep*. 2022;12(1):4645. Published 2022 Mar 17. doi:10.1038/s41598-022-08506-4

9.The length of the tPA used for the prediction of mRNA vaccine construct? Please mention it is it total length of 562 amino acids or part of it.

Response:Dear reviewer, thank you for your valuable suggestions.We used tPA signaling peptides to predict mRNA vaccine structure, but we only used its precursor sequence and not its total length. The tPA precursor sequence is a sequence that precedes the coding region of a structural gene, which can be transcribed but not translated.We have added lines 139-140 in the manuscript.

10.Figure4: Please mention the binding regions highlighted on the figure.

Response:Dear reviewer, thank you for your valuable suggestions.We have described the binding region in detail on lines 294-299.(Fig 4 B cell conformational epitope predicted by Ellipro.The yellow spheres represent B cell conformational epitopes,the gray area represents the majority of the polyprotein.(A)Rv0986 conformational epitope residues:**V228,N229,R230, E231,N232,Q233,T234,D235,Q236,P237,A238,S239,T240,I241,L242,L243,P244,T245,S246,Y247,E248.**(B)PknD conformational epitope residues:D242,S243,D244,R245,T246,T261,S262,L263, E264,H265,H266,H267.)

11.Please mention the reference or range for docking results like confidence score, RMSD

Response:Dear reviewer, thank you for your valuable suggestions.The higher the confidence score, the more reliable the interconnection result. The lower the RMSD value, the higher the protein stability. In our docking results, the confidence score is 0.9684 and the RMSD is 38.90A. Although there is no reference interval for the docking results at present, our docking results are very superior compared with published papers. The following is a reference:

(1).Yin Z, Li M, Niu C, et al. Design of multi-epitope vaccine candidate against Brucella type IV secretion system (T4SS). *PLoS One*. 2023;18(8):e0286358. Published 2023 Aug 10. doi:10.1371/journal.pone.0286358

(2).Shi J, Zhu Y, Yin Z, et al. In silico designed novel multi-epitope mRNA vaccines against Brucella by targeting extracellular protein BtuB and LptD. *Sci Rep*. 2024;14(1):7278. Published 2024 Mar 27. doi:10.1038/s41598-024-57793-6

12.Figure 6: Not visible to understand.

Response:Dear reviewer, thank you for your valuable suggestions.We have reuploaded Fig 6 and explained it in lines 309-311

13. Figure 7: Details are not matching with the text in 3.7. Some details are not clear EAAAK linker is missing in Schematic representation. In Scheme Author’s mentioned Epitopes as the linkers needs to be corrected like they are connected using linkers.

Response:Dear reviewer, thank you for your valuable suggestions.We are sorry for the error in Figure 7 due to our negligence. We have redrawn the scheme.

14. What is MCS in Figure B which indicated after the cloning sites (*Nhe*I AND *Pme*I) *Bam*HI and *Xho*I. It is not clear. All these details are very important for the design of the vector insertion of extra one nucleotide also changes the expression levels and their activity so authors need to be clearer regarding the sequence insertions while prediction.

Response:Dear reviewer, thank you for your valuable suggestions.MCS is a vector polyclonal site, which is a region of the vector itself.It is usually located downstream of the promoter(CMV). This region contains the restriction restriction sites BamHI,XhoI that we need. We need to insert the amplified target gene into this region. We have explained this in line 368-370.

15. Lane 321: Please correct the sentence it’s not the cloned sequence of PVAX1 was 2999bp. It’s just the vector size.

Response:Dear reviewer, thank you for your valuable suggestions.We have made changes in line 375.

16.Mentioned it clearly as vector size is 2999 bp, Insert Size is 1161 bp and the cloned construct size is 4160 bp. Why it is 4098?

Response:Dear reviewer, thank you for your valuable suggestions.Before inserting MEV-PCR, we first removed the two enzyme restriction site on the vector PVAX1, BamHI(730) and XhoI(786),a reduction of 57bp and selected two enzyme restriction site on MCS, which were located in BamHI(1) and XhoI(1156), an increase of 1156bp. PVAX1 was 2999bp, subtracted 57bp and added to 1156bp, resulting in 4098bp. For ease of understanding, we added the flowchart and named it Fig10A.The references are as follows：

(1).Fathollahi M, Fathollahi A, Motamedi H, Moradi J, Alvandi A, Abiri R. In silico vaccine design and epitope mapping of New Delhi metallo-beta-lactamase (NDM): an immunoinformatics approach. *BMC Bioinformatics*. 2021;22(1):458. Published 2021 Sep 25. doi:10.1186/s12859-021-04378-z.

(2).Motamedi H, Alvandi A, Fathollahi M, et al. In silico designing and immunoinformatics analysis of a novel peptide vaccine against metallo-beta-lactamase (VIM and IMP) variants. *PLoS One*. 2023;18(7):e0275237. Published 2023 Jul 20. doi:10.1371/journal.pone.0275237.

17.Figure 9D; what is Lane 2 and 3 and where the cloned plasmid of 4kb is. Please mention.

Response:Dear reviewer, thank you for your valuable suggestions.We have changed Figure9D to Figure10B and annotated Figure10 at 3.11. Lane 2 is MEV-PCR,Lane 3 is PVAX1. “1” stands for MEV-PCR,“2”stand for PVAX1,“3”stand for recombinant plasmid.

18.Figure 11: 3.13 if possible, The prediction of the secondary structure values needs to be compared with a standard

Response:Dear reviewer, thank you for your valuable suggestions.Due to the addition of figures, Figure11 has become Figure12.Protein secondary structures mainly include α helix, β sheet, random coil, and extended chain. The content of each structure varies with different proteins, so there is no standard reference value here. Considering your concerns about the structure of our vaccine, we detailed in the Quality testing of models of 3.14 that the vaccine we constructed in the Ramachandran Plot has 92.1% in the most favored regions, more than 90%, and 1.4% in the disallowed regions, less than 5%. Using ProSA-web, the Z-score was predicted to be -2.62, the energy diagramshows that most of the sequence positions are negative, all the above indicated that the vaccine structure we constructed was reasonable.

19.Lane 358: Interaction showed good characteristics. Please mention the range or the value obtained.

Response:Dear reviewer, thank you for your valuable suggestions.We have described the ranges or obtained values of RMSD,RMSF,hydrogen bond,Gyrate, and SASA in line 421-445.

20.Figure 14 not visible to understand.

Response:Dear reviewer, thank you for your valuable suggestions.Due to the addition of figures, Figure14 has become Figure15, and we have re-uploaded the figures according to the requirements of the magazine.

**Minor**

21. Grammatical Errors

Response:Dear reviewer, thank you for your valuable suggestions.We have corrected the grammatical problems in the manuscript.

22. Strain names should be in italics

Response:Dear reviewer, thank you for your valuable suggestions.We've italicized the strains.

23. Restriction enzymes name should be in italics

Response:Dear reviewer, thank you for your valuable suggestions.We have italicized the restriction restriction sites BamHI and XhoI.

**24.**Font variation between the sections

Response:Dear reviewer, thank you for your valuable suggestions.We have distinguished the font variations between the sections.

We tried our best to improve the manuscript and made some changes marked in yellow highlight in the revised paper which will not influence the content and framework of the paper. We appreciate for Editors and Reviewers’warm work earnestly, and hope the correction will meet with approval. Once again, thank you very much for your comments and suggestions.

With best wishes,

Yours sincerely.

Corresponding Author:Fengbo Zhang

fengbozhang@xjmu.edu.cn
