# Supplementary material for: Development of innovative multi-epitope mRNA vaccine against central nervous system tuberculosis using in silico approaches
Source: PLoS One. 2024 Sep 6;19(9):e0307877. doi: 10.1371/journal.pone.0307877 (PMC11379207; doi:10.1371/journal.pone.0307877)
Supplement: Attachment — Submitted filename: Reviewer comments.docx [file pone.0307877.s013.docx]

Manuscript number: PONE-D-24-15467

Decision: Minor revision.

General comments:

The authors of this study aimed to develop an innovative mRNA therapeutic vaccine targeting a protein associated with central nervous system tuberculosis, addressing the pressing need for effective treatments for this debilitating condition. Employing in silico techniques such as IEDB, NetCTLpan1.1, NetMHCIIpan-4.0, and SVMtrip, they analyzed the antigen epitopes of PknD and Rv0986. Additionally, CTL, HTL, and B cell epitopes were linked together using AAY, GPGPG, and KK linkers. This research was spurred by the lack of therapeutic options for central nervous system tuberculosis, underscoring its urgency.

The utilization of in silico methods paved the way for designing an mRNA therapeutic vaccine targeting central nervous system tuberculosis, offering a foundation for potential future experimental investigations. The outcomes of these assessments yielded promising observations, indicating the potential efficacy of the developed vaccine.

The authors have presented promising results in the manuscript. However, to optimize its effectiveness for our readers, it would be beneficial to address the following comments for streamlining and enhancement. While the manuscript exhibits good writing and organization, improvements in overall English language usage, particularly in grammar, punctuation, and clarity, would enhance engagement for general readers. Please consider revising the language to maximize effectiveness.

Comments:

1. Line 26: Define ‘BCG’ or provide more descriptive information as it appears first time here in this manuscript. Applicable for all abbreviations throughout the manuscript.
2. Line 33-34: please revise this sentence for effectiveness: ‘The results indicate that the vaccine structure shows promise’.
3. Line 40-41: The tense of the following sentence needs to be revised. If updated and most recent data is available, please provide that. ‘The World Health Organization (WHO) Global TB Report 2022 predicts that 41 10.6 million people will be diagnosed with TB in 2021, with 1.6 million fatalities.’
4. Line 42: Consider defining as this term is used many times in the manuscript ‘Central nervous system tuberculosis’.
5. Line 42: ‘TB’ is not defined, please define it.
6. Line 44: If authors could provide specifics in terms of specific mortality rate from the cited article, that would be helpful for readers.
7. Line 77 to 83: This paragraph has three references cited. This is confusing. Have these previous studies been conducted already? if they are, how they are related to the current study is not clear? please revise the language for clarity. If the intention is to cite for the part of the study, please make it clear.
8. Please revise the whole manuscript for formatting, such as: provide space between the last word in the sentence and the reference parenthesis. Applicable throughout the manuscript.
9. Line 87: link for the database should be before full stop. Please check the formatting throughout the manuscript.
10. 1All figures: Presently, all figures appear distorted and oddly stretched. Please recreate them with appropriate resolution and dimensions to ensure clarity. Additionally, consider increasing the font size on the figures for improved legibility. Some content on certain figures is currently illegible due to distortion. This feedback applies to all figures.
11. All section titles: provide a space between the number and section title, applicable for all section titles.
12. Validation of Models: It is advisable to elaborate on the validation process of the models utilized in this study to assess their reliability. Please address any limitations associated with validating these models to provide insight for readers or researchers interested in utilizing them in the future.
