# Supplementary material for: Development of innovative multi-epitope mRNA vaccine against central nervous system tuberculosis using in silico approaches
Source: PLoS One. 2024 Sep 6;19(9):e0307877. doi: 10.1371/journal.pone.0307877 (PMC11379207; doi:10.1371/journal.pone.0307877)
Supplement: Attachment — Submitted filename: Reviewer Comments _05-14-2024.docx [file pone.0307877.s015.docx]

**Development of innovative multi-epitope mRNA 2 vaccine against central nervous system tuberculosis 3 using in silico approaches**

Huidong Shi1 ,Yuejie Zhu2 , Kaiyu Shang1 , Tingting Tian1 , Zhengwei Yin1 ,Juan Shi1 ,Yueyue He3 4 ,Jianbing Ding3 ,Quan Wang4 *,Fengbo Zhang1 5 *

In this review, Shi et al, explained a in silico approach for the treatment of CNS Tuberculosis. The authors have explained a detail approach from the selection of the target sequences to codon optimization probabilities to ensure it’s translation in Human host. The authors needs to address these concerns

**Major:**

1. **All the figures are not clear/ visible to understand the results.**
2. Authors need to mention or provide the details of the length of the amino acids for each prediction like PknD (lenth:--a.a), Rv0986 (length :..a.a) which will be easily understandable for the reader when they are discussing about the signal peptide sequence or the % homologous sequences.
3. Figure 1: Schematic Representation of the flow of the work is not clear like after prediction of the signal peptide the authors are try to find the epitope sequences, it’s a continuous process but the authors mentioned as in prediction of signal peptide there are two different kind of stages like epitopes which is not correct. And in the later stages also. Please correct it.
4. Figure 2: MAFFT in Jalview is not clear enough to analyze.
5. In Lane 222: Authors mentioned the two proteins are highly homologous….What is the percentage of Homogeneity?. Please mention it.
6. In lane 231: Black parts means black bars or the black stretch or both which indicates the highly conserved amino acids. How many A.A/ how much percentage of the a.a are conserved. Please Mention it.
7. All the Figures are not clear so it’s very difficult to interpret the conclusion from the figures. (Example: Figure 8, nothing is visible to analyze the data).
8. Signal Sequence: Lane 130: tPA signal aids in secreting the signal sequence of epitope. In the results section authors mentioned there is no signal sequence detected in both the proteins. Please correct it,
9. The length of the tPA used for the prediction of mRNA vaccine construct? Please mention it is it total length of 562 amino acids or part of it.
10. Figure4: Please mention the binding regions highlighted on the figure.
11. Please mention the reference or range for docking results like confidence score, RMSD.
12. Figure 6: Not visible to understand.
13. Figure 7: Details are not matching with the text in 3.7. Some details are not clear EAAAK linker is missing in Schematic representation. In Scheme Author’s mentioned Epitopes as the linkers needs to be corrected like they are connected using linkers.
14. What is MCS in Figure B which indicated after the cloning sites (*Nhe*I AND *Pme*I) *Bam*HI and *Xho*I. It is not clear. All these details are very important for the design of the vector insertion of extra one nucleotide also changes the expression levels and their activity so authors need to be clearer regarding the sequence insertions while prediction.
15. Lane 321: Please correct the sentence it’s not the cloned sequence of PVAX1 was 2999bp. It’s just the vector size.
16. Mentioned it clearly as vector size is 2999 bp, Insert Size is 1161 bp and the cloned construct size is 4160 bp. Why it is 4098?
17. Figure 9D; what is Lane 2 and 3 and where the cloned plasmid of 4kb is. Please mention.
18. Figure 11: 3.13 if possible, The prediction of the secondary structure values needs to be compared with a standard
19. Lane 358: Interaction showed good characteristics. Please mention the range or the value obtained.
20. Figure 14 not visible to understand.

**Minor**

21. Grammatical Errors
22. Strain names should be in italics
23. Restriction enzymes name should be in italics
24. Font variation between the sections
